# Hippocampal and Left Subcallosal Anterior Cingulate Atrophy in Psychotic Depression

**DOI:** 10.1371/journal.pone.0110770

**Published:** 2014-10-22

**Authors:** Kelly Rowe Bijanki, Brendan Hodis, Michael C. Brumm, Emily L. Harlynn, Laurie M. McCormick

**Affiliations:** Department of Psychiatry, University of Iowa Roy J. and Lucille A. Carver College of Medicine, Iowa City, Iowa, United States of America; Banner Alzheimer’s Institute, United States of America

## Abstract

**Background:**

Psychotic depression is arguably the most diagnostically stable subtype of major depressive disorder, and an attractive target of study in a famously heterogeneous mental illness. Previous imaging studies have identified abnormal volumes of the hippocampus, amygdala, and subcallosal region of the anterior cingulate cortex (scACC) in psychotic depression, though studies have not yet examined the role of family history of depression in these relationships.

**Methods:**

20 participants with psychotic depression preparing to undergo electroconvulsive therapy and 20 healthy comparison participants (13 women and 7 men in each group) underwent structural brain imaging in a 1.5 T MRI scanner. 15 of the psychotic depression group had a first-degree relative with diagnosed affective disorders, while the healthy control group had no first-degree relatives with affective disorders. Depression severity was assessed with the Hamilton Depression Rating Scale and duration of illness was assessed in all patients. Automated neural nets were used to isolate the hippocampi and amygdalae in each scan, and an established manual method was used to parcellate the anterior cingulate cortex into dorsal, rostral, subcallosal, and subgenual regions. The volumes of these regions were compared between groups. Effects of laterality and family history of affective disorders were examined as well.

**Results:**

Patients with psychotic depression had significantly smaller left scACC and bilateral hippocampal volumes, while no group differences in other anterior cingulate cortex subregions or amygdala volumes were present. Hippocampal atrophy was found in all patients with psychotic depression, but reduced left scACC volume was found only in the patients with a family history of depression.

**Conclusions:**

Patients with psychotic depression showed significant reduction in hippocampal volume bilaterally, perhaps due to high cortisol states associated with this illness. Reduced left scACC volume may be a vulnerability factor related to family history of depression.

## Introduction

Depression is the highest contributor to disease burden in middle- and high-income countries [1], and among all of its subtypes, psychotic depression is the most severe variant [2], [3]. Psychotic depression represents a mood disorder distinct from non-psychotic major depression based on differences in symptomatology, disease course, treatment response, cognitive dysfunction, and biological features [4]. Approximately 20% of people with major depression are thought to experience psychosis, though this number rises to 50–60% in groups of patients with treatment-refractory major depression [5]. Patients with psychotic depression can be sensitively (84%) and specifically (99%) discerned from their non-psychotically depressed counterparts on the basis of their endorsements of unusual thought content, an item on the Brief Psychiatric Rating Scale [6], [7]. Patients with psychotic depression are reported to show greater cognitive deficits, have previous episodes with psychosis, longer duration of episodes, and a greater likelihood of recurrence [7]. In addition, they show greater hypothalamo-pituitary-adrenal axis activation and have higher cortisol levels than their non-psychotically depressed counterparts [8]. Psychotic depression has been advanced as the most diagnostically stable presentation of major depressive disorder [9]; in one study, 92.3% of patients with psychotic depression experienced previous and/or subsequent psychotic episodes [10]. From a research perspective, this diagnostic stability makes patients with psychotic depression an ideal group to study. Kronmuller and colleagues have elegantly articulated the importance of reducing variability in chronicity and severity of depressive illness for the purposes of research [11].

Reports of volumetric brain abnormalities in major depressive disorder have been mixed, likely owing to the heterogeneity of depressive symptoms and etiologies, as well as demographic differences among samples. Many studies report abnormalities of the hippocampus, amygdala, ventral anterior cingulate and prefrontal cortex in depressive illness [12]–[14]. Overall, it appears that there are significant effects of illness chronicity on hippocampus and amygdala atrophy in major depressive disorder, where longer illness duration correlates with more severe tissue loss [13], [15], [16]. One previous study [4] has examined hippocampus and amygdala volumes in three groups: psychotic major depression, non-psychotic major depression, and healthy controls. Results indicated that patients with psychotic depression had significant amygdala atrophy compared with the other two groups, and there were no significant group differences in hippocampal volumes. In contrast, a more recent study [50] examined virtually identical participant groups and found no significant differences between psychotic and non-psychotic major depressive disorder patients in amygdala or hippocampus volumes. However, they did observe that patients with major depression (regardless of the presence of psychosis) tended to have larger amygdala volumes than controls.

In recent years, research on depression has turned toward the ventral anterior cingulate cortex and the two sub-regions that comprise it; the subcallosal and subgenual regions (scACC and sgACC respectively). Decreased activity has been noted in these areas in response to antidepressant medication, placebo treatment, and electroconvulsive therapy (ECT). Increased activity in these regions is noted in treatment non-responders, the currently depressed and transiently sad people compared to healthy controls [17]. One recent study [50] examined group-wise differences in the volume of this brain region in patients with psychotic depression as compared with non-psychotic major depression and healthy controls, reporting no significant differences between depression groups, but significant atrophy in the left subgenual region compared with controls.

**Figure 1:**
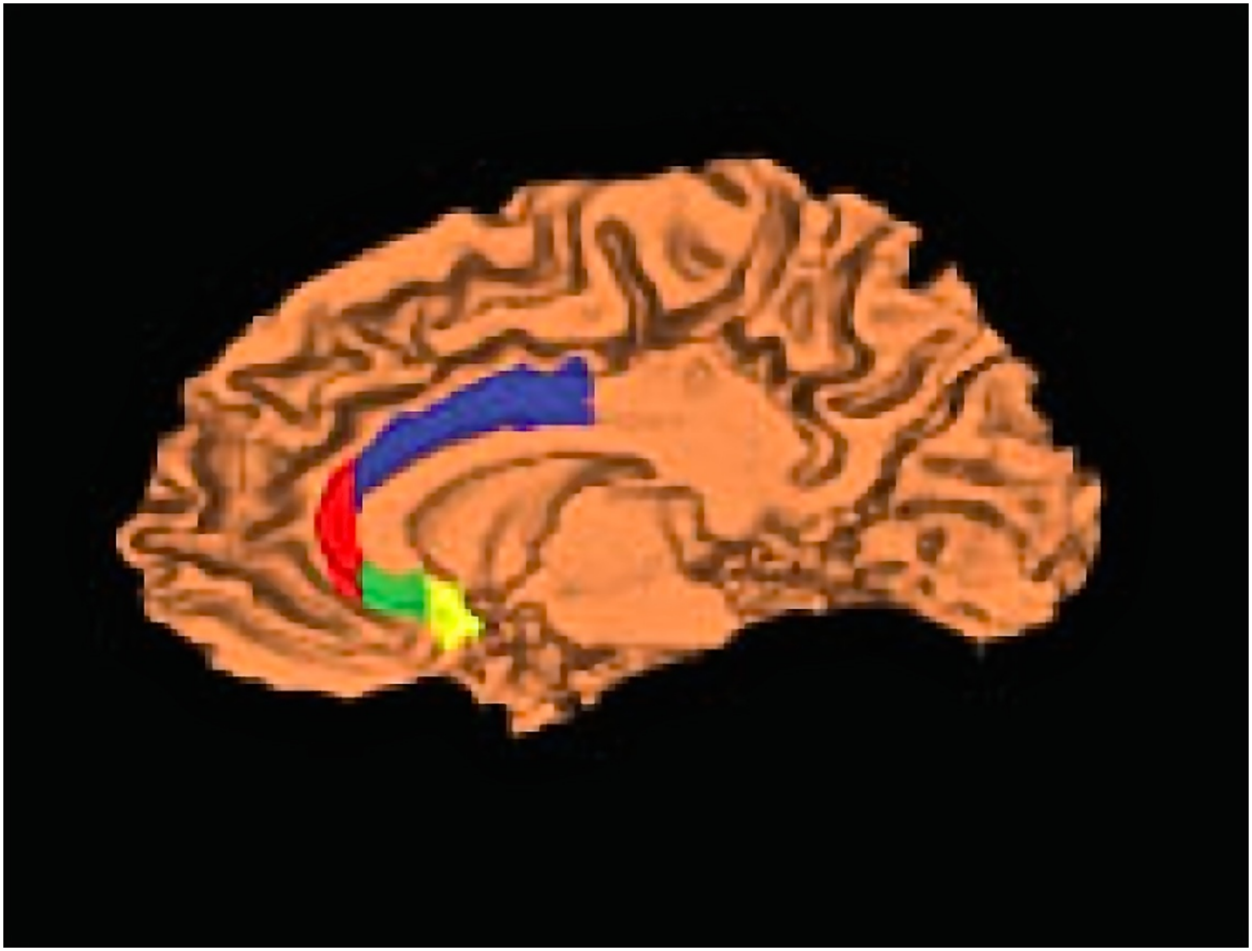
Parcellations of the anterior cingulate cortex. Dorsal anterior cingulate cortex (ACC) shown in blue, rostral ACC in red, subcallosal ACC in green, subgenual ACC in yellow. Figure reprinted with permission from John Wiley and Sons, from McCormick et al., (2008), license number 3103240766914.

An important terminological shift must be acknowledged in the literature on the ventral anterior cingulate. Early research on the anatomy and function of the area used the term “subgenual” to define the entirety of the ventral anterior cingulate cortex underlying the genu of the corpus callosum. In recent years, the term “subgenual” has been revised to include only a small volume of the posterior ventral ACC, also frequently termed “Brodmann Area 25” (Figure 1, yellow region). The anterior section of the ventral ACC, or ventral Brodmann Area 24 has since been termed “subcallosal” (Figure 1, green region). For example, decreased activity has been shown in the “subgenual cingulate” (identical to the subcallosal + subgenual cingulate as defined in the current study) in patients with familial unipolar major depressive disorder and that this decrease in activity corresponded with a 48% reduction in volume of the structure [18].

In the current study we aimed to examine the neural correlates of psychotic depression using structural MRI volumetric techniques. Based on findings reported across the depression literature, we defined six regions of interest for the current study: the hippocampus, the amygdala, and four parcellations of the anterior cingulate cortex (dorsal, rostral, subcallosal, and subgenual). Two previous reports from our lab [19], [20] have suggested the left subcallosal cingulate and the hippocampus as major mediators of treatment response in ECT for psychotic depression. We anticipated seeing group differences in volumes in these regions, especially on the left side. In contrast with the previous study by Vassilopoulou and colleagues, we also aimed to examine the role of family history of affective disorders in any observed volumetric abnormality of these structures. Furthermore, the current study expands on previous findings [4] by including measures of the anterior cingulate cortex and by examining patients whose depression may have been more homogeneous (in that all participants in the current study experienced very severe psychotic depression and were being treated with ECT at the time of neuroimaging). The current study additionally expands on the findings of Drevets et al. [18] by examining patients with psychotic depression and including measures of medial temporal lobe structures.

## Methods

### Participants

The current study included 20 participants with psychotic unipolar major depression (here termed “psychotic depression”) and 20 age- and sex-matched healthy comparison participants (see Table 1 for demographics). Healthy comparisons were recruited from the community through newspaper advertisements and were screened using an abbreviated version of the Comprehensive Assessment of Symptoms and History. Comparison participants were excluded if they had current or past major medical, neurological, or psychiatric illnesses, or if they had a family history of schizophrenia or affective disorders.

**Table 1:** Demographic Comparison between Psychotic Depression and Healthy Control groups.

	Psychotic DepressionMean (SD) [n = 20]	HC Mean (SD) [n = 20]	DF	Statistic	*p*-value
Age	46.0 (9.5)	45.4 (9.9)	38	t = −0.201	*p* = 0.841
Education	13.3 (2.8)	14.5 (1.6)	38	t* = *1.737	*p* = 0.090
Sex	65% F (13 f: 7 m)	65% F (13 f: 7 m)	1	*X* ^2^ = 0.000	*p*>0.999
HAM-D	38.5 (5.4)	–	–	–	–
HAM-A	28.8 (7.9)	–	–	–	–
Family History ofAffective Disorders	75%	–	–	–	–
Medication Use					
Antidepressant	95%	–	–	–	–
Anxiolytic	25%	–	–	–	–
Antipsychotic	40%	–	–	–	–
Lithium	5%	–	–	–	–

Note: Age and education reported in years. HAM-D = Hamilton Depression Rating Scale, HAM-A = Hamilton Anxiety Rating Scale, HC = healthy control, DF = degrees of freedom, SD = standard deviation. Family history of affective disorders included evidence of depression, bipolar disorder, or suicide attempt in a first degree relative. Data on family histories of affective disorders and medication usage were taken from interviews conducted by trained psychiatrists during the course of treatment and documented in the participants’ medical records. Medications were assessed an average of 8 days prior to MRI scan and excluded “pro re nata” medications given for acute care of psychotic depression during hospitalization.

The participants in the psychotic depression group took part in a larger study that reported neuroimaging findings before and after ECT [19], [20]. All participants met DSM-IV criteria for major depression with psychotic features and completed an MRI scan prior to or early in treatment with ECT (scans gathered after an average of 3.5 ECT doses). Eighteen participants (90%) underwent inpatient hospitalization at the University of Iowa Hospitals and Clinics and were referred for ECT by their psychiatrist on clinical grounds; the other two participants (10%) had been referred for ECT treatment on an outpatient basis. Data on the duration of depressive episode, as well as family histories of affective disorders and information on medication usage (Table 1) were taken from interviews conducted by trained psychiatrists during the course of treatment and documented in the participants’ medical records. Participants had a range of current episode duration: 55% had been depressed for less than 6 months; 20% had been depressed for between 6 and 12 months; 5% had been depressed for between 12 and 24 months; 20% had been depressed for greater than 24 months at the time of data collection. Participants were rated as having a family history of affective disorder if there was unequivocal documentation of a first-degree relative (i.e. parent, sibling, or child) with diagnosed depression, bipolar disorder and/or suicide attempt based upon review of their medical records. Medications were assessed an average of 8 days prior to MRI scan and excluded “pro re nata” medications given for acute care of psychotic depression during hospitalization.

All participants in the psychotic depression group had a pre-ECT score of at least 30 on the 28-item Hamilton Depression Rating Scale (indicating severe depression) and had at least one psychotic symptom. Participants were excluded if they had a diagnosis of bipolar disorder or schizophrenia, met criteria for alcohol or illicit drug abuse or dependence within the past three months, or had been treated with a previous course of ECT within the past three months. After complete description of the study to the participants, written informed consent was obtained. This study was approved by The University of Iowa Institutional Review Board (200402013).

### Measures

All participants in the psychotic depression group completed clinical interviews using the Scales for the Assessment of Negative and Positive Symptoms (SANS/SAPS) [21], [22]. Scores from the SANS/SAPS were divided into three dimensions: (1) psychoticism (scale 0–10), based on global ratings of delusions and hallucinations; (2) disorganization (scale 0–15), based on global ratings of bizarre (disorganized) behavior, positive formal thought disorder, and inappropriate affect; and (3) negative symptoms (scale 0–25), based on global ratings of alogia, anhedonia-asociality, avolition-apathy, affective flattening, and attention. The most common psychotic symptom in the current sample was delusional guilt.

In addition, all psychotic depression participants completed the 28-item Hamilton Depression Rating Scale (HAM-D) [23], as well as the Hamilton Anxiety Rating Scale (HAM-A) [24]. Both are clinician-administered instruments that assess symptoms and severity of a patient’s depressive and anxious symptoms, with higher scores indicating increasing severity of symptoms. Scores above 30 on the HAM-D and HAM-A are generally taken to indicate “very severe” anxiety and depression.

### Neuroimaging

Multimodal magnetic resonance imaging (MRI) scans were obtained using either a 1.5-T GE Signa scanner (10 psychotic depression participants and 20 healthy controls; GE Medical Systems, Milwaukee, WI) or on a 1.5-T Siemens Avanto scanner (10 psychotic depression participants; Erlangen, Germany). The sequences collected included a standard T1-weighted three-dimensional spoiled gradient recall acquisition (TE = 5 ms; TR = 24 ms; flip angle = 30°−50°; two excitations; FOV = 26 cm; matrix = 256×256; slice thickness = 1.5–1.7 mm). Two-dimensional T2 sequences were also acquired (TE = 24–98 ms; TR = 3000–9500 ms; flip angle = 90°−180°; one excitation; FOV = 26 cm; matrix = 256×256; slice thickness = 3.0 mm). All scans were visually assessed for quality and for motion artifacts.

Automated processing of the structural images was performed using BRAINS2 (Brain Research: Analysis of Images, Networks, and Systems) software [25], which includes automated AC-PC alignment, image alignment, image intensity standardization, tissue classification, and brain extraction. Then, automated neural nets were used to parcellate the hippocampi and amygdalae bilaterally [26], [27]. These neural nets were then reviewed by human raters following procedures as outlined by [28] and, when necessary, hand-corrected following procedures as outlined by [29].

The hand-tracing methods for parcellating the ACC (Figure 1) have been described at length elsewhere [30], and the previous methods were strictly followed for the identification of the dorsal, rostral, and subgenual ACC. For the current study, the previous parcellation method for the subcallosal ACC was modified slightly; the subcallosal region includes only the continuation of the rostral cingulate gyrus as it wraps around the genu of the corpus callosum, rather than additionally including paracingulate gyrus. The anterior boundary of the subcallosal ACC is defined by the anteriormost slice where the forceps major of the corpus callosum is no longer connected through the genu on the coronal view. The posterior boundary is placed one slice anterior to where the putamen first is visible within the basal ganglia. The superior boundary is formed by the ventral continuation of the callosal sulcus. The inferior boundary of this region is defined by the termination of the primary cingulate sulcus (whether or not a secondary sulcus is evident) [31].

Twenty-one psychotic depression participants and 20 HC participants received structural neuroimaging scans. One psychotic depression participant was excluded from the study due to profound damage from a traumatic brain injury. Two other psychotic depression participants’ scans failed the amygdala and hippocampus neural net application and were excluded from those analyses, though their ACC regions were parcellated satisfactorily. One HC participant’s scan failed the amygdala ANN but his or her data were usable for hippocampal and ACC measures. Therefore, the following samples were available for analysis: amygdala (18 psychotic depression, 19 HC), hippocampus (18 psychotic depression, 20 HC), ACC subregions (20 psychotic depression, 20 HC). Of the two psychotic depression participants whose amygdala and hippocampus neural nets failed, one had a positive family history of affective disorders.

### Statistical Analyses

All data underlying the current manuscript are presented as Supplementary Files (Dataset S1, Dataset S2, Variable Definitions S1), including raw numerical data and variable descriptions. Statistical analyses used the standard alpha level of .05 for statistical significance and used two-tailed tests. Demographic data were examined to demonstrate that there were no significant differences between the experimental and control groups, using independent samples t-tests (age, education) and chi-squared tests (sex) (Table 1). For methodological reasons, half of the psychotic depression group received their neuroimaging scans on a different scanner (Siemens) than the rest of the participants (GE). For this reason, we used a multivariate general linear model to confirm that there were no effects of scanner on the imaging measures used in the study.

Next, independent samples t-tests were used to examine the volumetric differences in the regions of interest (hippocampus, amygdala, dorsal ACC, rostral ACC, subcallosal ACC, subgenual ACC, and total intracranial volume) between the experimental and control groups. The hippocampus test precluded an assumption of equal variances, so the degrees of freedom for that test were adjusted accordingly. After the initial hypothesis testing, these group-wise comparisons were re-calculated using a multivariate model adjusting for total intracranial volume (indicated with asterisks in Table 2). Finally, the results of these comparisons were further adjusted for the false discovery rate [32], and results remaining significant after that adjustment are indicated with pluses (Table 2).

**Table 2:** Comparison of psychotic depression group with healthy controls on brain volumetrics.

	Psychotic Depression Mean (SD)	HC Mean (SD)	DF	Statistic	*p*-value
Total ICV	1345.74 (170.18)	1388.78 (160.59)	38	t = 0.823	*p* = 0.416
Hippocampus^a^	4.12 (0.37)	4.58 (0.52)	34.33	t = 3.166	*p* = 0.003* +
Amygdala^b^	2.18 (0.30)	2.28 (0.36)	36	t = 0.948	*p* = 0.349
Subcallosal ACC	0.71 (0.23)	0.93 (0.22)	38	t = 3.036	*p* = 0.004* +
Subgenual ACC	1.02 (0.28)	1.04 (0.29)	38	t = −0.196	*p* = 0.845
Dorsal ACC	7.39 (1.26)	7.00 (0.88)	38	t = −1.139	*p* = 0.262
Rostral ACC	4.32 (1.66)	4.20 (1.04)	38	t = −0.263	*p* = 0.794

Note: Raw means and standard deviations presented (reported in cubic centimeters).

*comparisons that remain significant after adjustment for total ICV.

+ comparisons that remain significant after adjustment for false discovery rate.

a sample included 18 psychotic depression and 20 HC participants with no significant differences between groups on demographic variables (age *p* = 0.772, education *p* = 0.114, sex *p* = 0.804).

b sample included 19 psychotic depression and 19 HC participants. Hippocampal t-test adjusted because of violation of the assumption of equal variances. ICV = intracranial volume, reported in cubic centimeters. ACC = anterior cingulate cortex.

A more detailed examination of laterality effects in the hippocampus and subcallosal ACC was conducted using independent samples t-tests and repeating the statistical adjustments for total intracranial volume and false discovery rate. Finally, we examined the psychotic depression group to see if there was an effect of family history of affective disorders on hippocampal and subcallosal ACC volumes, again using independent samples t-tests.

## Results

There were no significant differences between participants with psychotic depression and healthy comparisons in terms of sample size, age, education, or sex (see Table 1). Patients with psychotic depression additionally completed the Hamilton Depression and Anxiety Rating Scales (HAM-D and HAM-A) (Table 1), as well as Scales for the Assessment of Negative and Positive Symptoms of Schizophrenia (SANS: mean = 12.9, SD = 3.4; SAPS-Psychoticism: mean = 3.3, SD = 1.8; SAPS-Disorganization: mean = 0.5, SD = 0.8). The psychotic depression group was further characterized in terms of family history of affective disorders, where fifteen of the twenty participants with psychotic depression had a family history of a first-degree relative with an affective disorder. There were no differences between psychotic depression patients with and without family histories in terms of baseline HAM-D score (t = −.065, DF = 19, *p* = .949). Psychotic depression patients had an average duration of current episode of less than six months (55%), and the majority used antidepressant medications (Table 1). Because of the possibility of confounding due to the use of two different scanners in the neuroimaging acquisition, we used a multivariate general linear model to confirm that there were no effects of scanner on the imaging measures used in the study. This analysis confirmed that there was a non-significant effect of scanner on the brain regions examined in the current study (Pillai’s Trace = 0.315, F = .880, *p* = .577).

Group-wise comparisons examined regional brain volumetric differences between psychotic depression and HC participants in raw volumes of the hippocampus, amygdala, dorsal ACC, rostral ACC, subcallosal ACC, and subgenual ACC. These analyses showed that the only significant differences between groups were in the hippocampus and the subcallosal ACC (see Table 2). These comparisons were calculated twice; once uncorrected, and once controlled for total intracranial volume and adjusted for false discovery rate.

Laterality effects were then assessed. While both sides of the hippocampus were found to be significantly smaller in psychotic depression participants than healthy comparisons, the reduction in subcallosal ACC volume was driven primarily by left-sided atrophy (Table 3). Hippocampal volume in the psychotic depression group averaged 10.1% smaller than healthy controls, while the left subcallosal ACC was 31.4% smaller than controls. Furthermore, it was found that within the psychotic depression group, those with a positive family history of affective disorders (15 of the 20 participants) had significant atrophy of the left subcallosal ACC (t = 3.809, df = 24.590, *p* = .001) when compared with healthy controls, while the five without family history showed normal volume of the structure (t = .497, df = 23, *p* = .624). On average, psychotic depression patients with a family history of affective disorders had left scACC volumes 38.5% smaller than healthy controls. By comparison, there was no effect of family history of affective disorder on the finding of hippocampal atrophy (t = .531, df = 16, *p* = .603). Both subgroups of depressed patients had significantly smaller hippocampal volumes than healthy comparison subjects (positive family history: t = 2.350, df = 10.888, *p* = .039; negative family history: t = 2.972, df = 32, *p* = .006).

**Table 3:** Laterality effects on hippocampus and subcallosal ACC volumes in psychotic depression compared to age-matched healthy comparisons.

	Psychotic Depression Mean (SD) [n = 20]	HC Mean (SD) [n = 20]	DF	Statistic	*p*-value
Left Hippocampus^a^	2.05 (0.181)	2.30 (0.276)	33.08	t = 3.303	*p* = 0.002 * +
Right Hippocampus^a^	2.07 (0.214)	2.28 (0.262)	36	t = 2.797	*p* = 0.008 * +
Left subcallosal ACC	0.289 (0.087)	0.421 (0.174)	38	t = 3.035	*p* = 0.004 * +
Right subcallosal ACC	0.425 (0.213)	0.507 (0.143)	33.25	t* = *1.420	*p* = 0.165

Note: Raw means and standard deviations presented (reported in cubic centimeters).

*comparisons that remain significant after adjustment for total ICV.

+ comparisons that remain significant after adjustment for false discovery rate.

a sample included 18 participants with psychotic depression and 20 HC participants with no significant differences between groups on demographic variables (age *p* = 0.772, education *p* = 0.114, sex *p* = 0.804). Left hippocampal and right subcallosal ACC t-tests adjusted because of violation of the assumption of equal variances. ICV = intracranial volume, reported in cubic centimeters. ACC = subcallosal anterior cingulate cortex.

## Discussion

Participants with psychotic depression were compared with healthy controls on measures of the hippocampus, amygdala, dorsal ACC, rostral ACC, subcallosal ACC, and subgenual ACC. As hypothesized, this study demonstrates a pattern of significantly smaller bilateral hippocampus and left-sided subcallosal cingulate volumes in patients with psychotic depression when compared to age-matched healthy control participants. Left subcallosal ACC atrophy was highly significant only in the group of psychotic depression subjects with a family history of affective disorders, while hippocampal atrophy was found bilaterally and was not dependent on whether there was a family history of affective disorders.

Left scACC atrophy in patients with depression who also have a family history of affective disorders is consistent with three previous studies, all of which examined the undifferentiated “subgenual” region, which included what are now termed the subcallosal and subgenual subregions [18], [33], [34]. The current finding is consistent with that of [18], which reported a 48% reduction in left “subgenual” cingulate volume in a group of patients with familial unipolar depression compared with healthy controls. The second study [33] showed a 20% reduction in left “subgenual” cingulate volumes in patients with affective disorders compared with healthy controls, and a 24% reduction in patients with familial affective disorders compared to patients with non-familial affective disorders. The third study [34] further showed that women with MDD had significantly smaller left “subgenual” ACC volumes than controls, with no reduction on the right. Another study has shown a 23.8% reduction in the volume of the subgenual cingulate bilaterally in familial major depressive disorder [35]. Histological analysis showed that this volume reduction was related to decreased numbers of glia in the region while the number of neurons remained unchanged [35]. It is unclear why only the left sided atrophy of the subcallosal cingulate cortex appears to be driven by genetics in the current sample, in that it was only observed in those psychotic depression patients with a family history of affective disorders. However, one previous study [50] in patients with psychotic depression reported a similar finding, and similarly constrained to the left subgenual cingulate.

Several previous studies have identified the presence of bilateral hippocampal atrophy in MDD patients, which was supported by the findings of the current study [12], [13], [15], [16]. We did not confirm the previous finding from Keller et al. [4], who reported amygdala atrophy rather than hippocampal atrophy specifically in psychotic depression. This discrepancy could be related to the use in the current study of a new, automated method for measuring amygdala and hippocampus volumes, rather than the manual method used in the previous study, which was originally developed for use in a pediatric population [36]. The current study also used a 1.5 T MRI (3T in Keller et al.), as well as older (mean age 45 in the current study; 36 in Keller et al.), and more depressed participants (mean HAM-D = 38 current study; mean HAM-D = 30 in Keller et al.).

The exact neurobiological underpinnings of depression and psychotic depression remain to be completely elucidated, though there are hints at the mechanisms, one of which is related to glucocorticoid toxicity of the hippocampus following prolonged exposure to the stress hormone cortisol. Intense stress, such as that experienced during severe depression, can cause reduced expression of brain-derived neurotrophic factor (BDNF) in the hippocampus and elevated expression of cortisol, leading to decreased hippocampal neurogenesis and overall atrophy of the structure [37], [38]. Patients with psychotic depression may be especially vulnerable to decreased hippocampal neurogenesis and overall atrophy because they (more so than their non-psychotic depressed counterparts) experience elevated cortisol expression [4], [39] ECT, and to a lesser extent antidepressant medications, have been shown to promote synaptogenesis and neurogenesis in the hippocampus [40]–[42]. Psychotic depression is highly responsive to ECT treatment, with a 95% response rate [43] and the mechanism of this response may be linked to increased neurogenesis in the hippocampus [42]. It is possible that in psychotic depression, ECT is the most effective treatment because it most strongly promotes neurogenesis in the hippocampus, perhaps even to such an extent that it can overcome the stress-induced atrophy of the structure.

The ACC and the hippocampus are connected through the limbic system and may function as pacemakers for theta oscillations in the brain [44]–[46]. Previous studies have shown that theta activity in the ACC could predict treatment response to antidepressants, and that ECT could change theta activity in the scACC in depressed patients [47], [19]. The scACC is also the primary brain target for deep brain stimulation (DBS) for treatment-resistant depression in a large multi-site study [48]. Resolution of depressive symptoms with DBS of the scACC has also been associated with changes in theta activity and pre-treatment theta activity may even predict response to DBS [49].

The current study included a sample of patients with psychotic depression that was so severe and treatment resistant to necessitate treatment with ECT. The current study was limited by a relatively small sample, a lower-powered MR scanner, and included only cross-sectional data. In order to remedy the limitations of the current study, future studies with this population should include both pre- and post-ECT MRI with a higher resolution 3T scanner and with diffusion weighted imaging to allow tractography studies to specifically look at hippocampal-ACC connections. It will also be important to further assess whether these findings are specific to psychotic depression or high cortisol states, and whether they exist in other subtypes of MDD severe enough to require ECT. However, the current study has shown for the first time that the volume of the hippocampus is significantly smaller in patients with psychotic depression than in controls, and that there are significant effects of family history on atrophy of the scACC specifically. These findings may provide further insight into the pathophysiology of psychotic depression, and highlight the importance of consideration of family history and brain structural changes over the course of the illness.

## Supporting Information

Dataset S1 **All data underlying the current manuscript are presented here in comma-separated variable format for easy access.**
(CSV)

Dataset S2 **All data underlying the current manuscript are presented here in IBM SPSS software file format with variable definitions and other descriptive information embedded.**
(SAV)

Variable Definitions S1 **Definitions for each variable heading in Dataset S1 and Dataset S2.**
(CSV)
